# The Difference

**Published:** 1885-11-20

**Authors:** 


THE DIFFERENCE.—We have before alluded to the extreme economy of the Atlanta Municipal Government in regard to the estimate placed upon medical services. In Atlanta, the City Physician gets about 87 cents a day, and furnishes his own drugs! In Charleston, we are credibly informed the city physician is paid $1,000 per annum, with the addition of $250 for his horse, and his drugs also furnished. This, in view of the extent and disagreeable character of the practice required, is low enough certainly; yet, as compared with the compensation of the Atlanta city physician's, it is princely.

